# Evaluation of bone healing in trabeculae structure of mandibular corpus and angulus fracture patients with fractal dimension analysis

**DOI:** 10.1016/j.jobcr.2024.12.015

**Published:** 2025-01-25

**Authors:** Poerwati Soetji Rahajoe, Hendra Sukawijaksa, Pingky Krisna Arindra, Silviana Farrah Diba

**Affiliations:** a Department of Oral and Maxillofacial Surgery, Universitas Gadjah Mada, Yogyakarta, Indonesia; b Department of Dentomaxillofacial Radiology, Universitas Gadjah Mada, Yogyakarta, Indonesia

**Keywords:** Corpus fracture, Angulus fracture, ORIF, fractal dimension, Trabeculae pattern

## Abstract

**Background:**

Fractures of the mandibular corpus and angulus are affected by different forces result from different strength of the muscles that influenced them. Therefore, it is presumed that there is a different in healing rate. Fractal dimension radiologic analysis assesses the trabecular pattern, due to it can be used to observe the healing process of bone fractures.

**Purpose:**

The study aimed to compare the healing process of mandibular corpus and angulus fractures with fractal dimension analysis (FD) of trabecular patterns on panoramic radiographs through ImageJ software as consideration to determine mastication loading time.

**Methods:**

This retrospective cross-sectional study observed medical records and panoramic radiographs of patients with a diagnosis of corpus and or mandibular angulus fractures undergoing open reduction (ORIF) 1 plate treatment combined with maxillomandibular fixation (MMF) at Hospital in 2017–2023. Observations were carried out on postoperative 1st day, 2nd week, 8–13th week and >13th week respectively.

**Results:**

Twenty-five subjects who met the inclusion requirements were involved in the study. The results of Two-Way ANOVA statistical test and Fisher's LSD post-hoc showed that the FD values of corpus fracture were significantly greater than that of mandibular angulus with p 0.000 (H1), 0.003 (H2), 0.03 (H3), 0.000 (H4) respectively. In both groups of mandibular corpus and angulus fractures, there was a significant effect of time with p = 0.00 in each group. The longer the observation time, the more trabeculae patterns were formed in the healing process of corpus and angulus fractures.

**Conclusion:**

Post ORIF observation of corpus fracture shows a greater trabecula formation value which indicates faster healing when compared to mandibular angulus fracture.

## Graphical abstract

**Figure:**
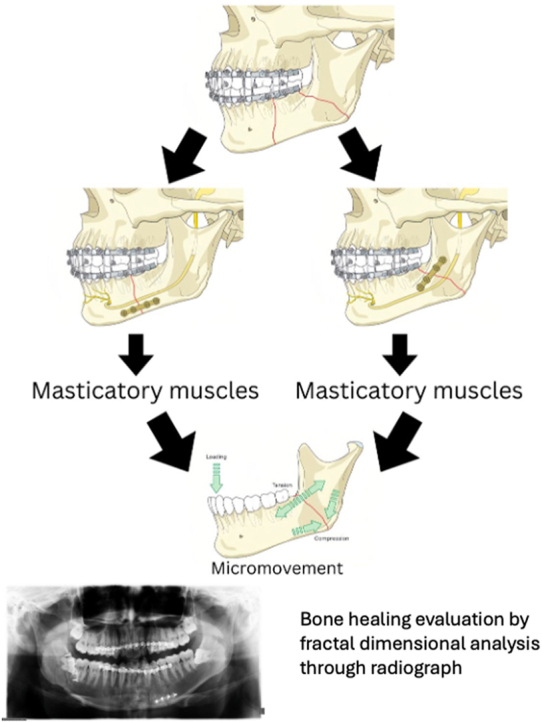

## 1 Introduction

One of the treatment choices for mandibular corpus and angulus fractures is Open Reduction Internal fixation (ORIF).^1^ Open reduction will result in better visibility and access for reduction and repositioning of the fracture area. Furthermore, inserted plates and screws will bring about more rigid fixation between fracture fragments.^1^^,^^2^ The additional intermaxillary fixation sometimes needed to promote rigidity and will be removed in about 1–2 weeks based on clinical conditions.^3^ The MMF procedure aimed at maintaining proper occlusal relationship by fastening the teeth, while stabilizing the fracture segment.^4^ The treatment depends on the condition and indication of each case, such as fragment displacement, malocclusion severity, fracture line complexity, and fracture position advantage.^2^

Post ORIF of mandibular fractures, the movement of the main masticatory muscles in the angulus and accessories in the corpus can produce 2 forces: tension (pull) and compression (pressure) on the fracture line.^5^ The difference in bone healing speed of corpus and angulus fractures has never been studied. Fractures of the angulus region are influenced by the main masticatory muscles including masseter, temporal, medial pterygoid, and lateral pterygoid, which will have the effect of pulling the proximal segment to the superior and medial. Mandibular corpus area is dominated by the movement of fracture fragments of accessory masticatory muscles, such as digastricus and genio-digastric muscles in pulling the mandible inferiorly, causing the segment to shift.^5^, ^6^, ^7^ At the corpus mandibular side, tension occurs in the area along the alveolar teeth and compression takes place in the area along the inferior margo of the mandible, while at the angulus mandible, tension will occur in the superior border area and compression in the inferior border of the mandible when mastication loading occurs in the anterior area. On the other hand, if mastication loading occurs in the posterior area, tension occurs in the inferior area and compression in the superior border of the mandible.^8^ The release of MMF allows jaw mobility due to functional movement. It may result in micro-movement between bone fragments, because of the difference in tension and compression forces during mastication function. Therefore, it is likely to cause different healing speed of mandibular fractures in the angulus and corpus areas.^5^

The inspection of bone healing is commonly done by checking the mobility between fragments.^9^^,^^10^ The radiographic examination, however, is used as an additional evaluation of bone healing by observing changes in the area towards radiopaque qualitatively on the fracture line. Therefore, a quantitative imaging analysis is needed to observe the bone healing process.^11^^,^^12^ Fractal dimension analysis is one of the image analyses to assess the structure of the trabeculae pattern so as to quantitatively assess the bone healing process.^13^^,^^14^

Several studies were conducted to observe the bone healing process with fractal dimension analysis. Heo et al. (2002), Kang et al. (2012) and Colak et al. (2022) evaluated the bone healing process through fractal dimension analysis in Bilateral Sagittal Split Osteotomy patients.^13^^,^^15^^,^^16^ Coban et al. (2022) evaluated bone healing of osteotomy patients in genioplasty.^17^ The results of Heo et al. (2002), Kang et al. (2012), Colak et al. (2022) and Coban et al. (2022) showed that there was an increase in the fractal dimension value in the osteotomy area as the bone healing process occurred.^13^^,^^15^, ^16^, ^17^ The study which used the fractal dimension analysis to observe the fracture healing of the corpus and mandibular angulus has never been carried out. This study aimed to compare the bone healing process of mandibular corpus and angulus fractures with fractal dimension analysis (FD) of panoramic radiographic trabeculae patterns through ImageJ software.

## 2 Material and methods

This crosssectional retrospective study involved samples of panoramic radiographic photographs of subjects/patients with a diagnosis of mandibular corpus and or angulus fractures who underwent Open Reduction Internal Fixation (ORIF) surgery at RSUD from 2017 to 2023. Ethical eligibility of the study was approved by the ethics committee of Faculty of Dentistry of the University (32/UN1/KEP/FKG-RSGM/EC/2024).

Inclusion criteria include medical record data of patients with fractures of the mandibular corpus or angulus, ORIF surgery with 1 piece of fixation plates with a minimum of 4 couplers attached to each fracture area, medical record data and complete postoperative panoramic radiographs (based on the specified observation time), patients had no systemic diseases, aged 18–60 years. Exclusion criteria include comminuted fractures, open fractures, post ORIF complications such as infection, dehiscence, open plate, radiology image quality ineligible for fractal dimension analysis as assessed by a experienced radiologist (SFR). The radiograph images were taken using the same panoramic radiography device as ®Instrumentarium OP300 Panorex, radiation exposure factors: tube voltage 66 KVP, tube current 9.9 mA, exposure time 14.1 s and beam irradiance 54.4 mGy cm^2^).

### 2.1 Research sampling

Medical record data and OPG radiographs of patients who met the inclusion criteria were recorded. Recording clinical data include the characteristics of the subjects such as age, gender, direction of fracture line, number of molar teeth, education level, nutrition status and duration of MMF. It also covers a follow-up evaluation of patient conditions: pain scores, and complications that occurred (paresthesia, dehiscence, infection, malocclusion, fragment displacement). Radiological data include postoperative observation times on postoperative 1st day (H1), 2nd -3rd week (H2), 8–13th week (H3), and >13th week (H4) and their respective FD values. Selected panoramic radiograph images were subjected to Region of Interest (ROI) determination and fractal dimensional analysis.

### 2.2 Determination of Region of Interest (ROI)

**Fig. 1:**
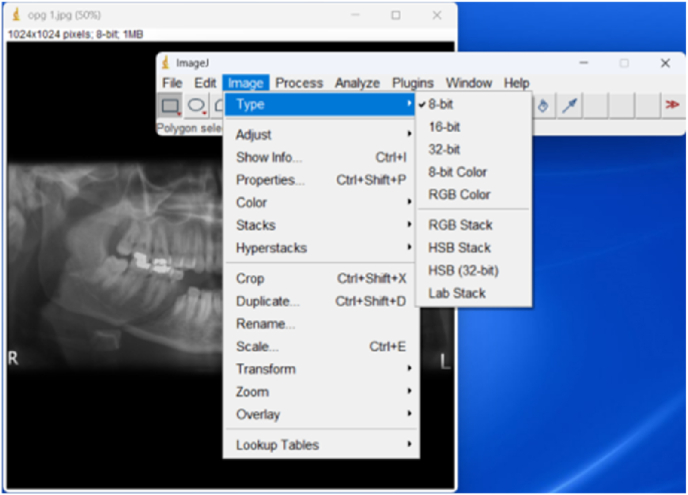
Adjusted Panoramic radiograph type in ImageJ.

Region of Interest was determined by one experienced radiologist (SFR), who performed the Intraobserver Intraclass Correlation Coefficient (ICC) Test with a value of 0.89. Images in Image-J software of panoramic radiographs were first equalized in pixel size with a pixel size of 930 × 434 pixels and then converted into 8-bit image data (Fig. 1). The conversion menu in Image-J software was used to make sure that panoramic radiographs have the same pixel size.

**Fig. 2:**
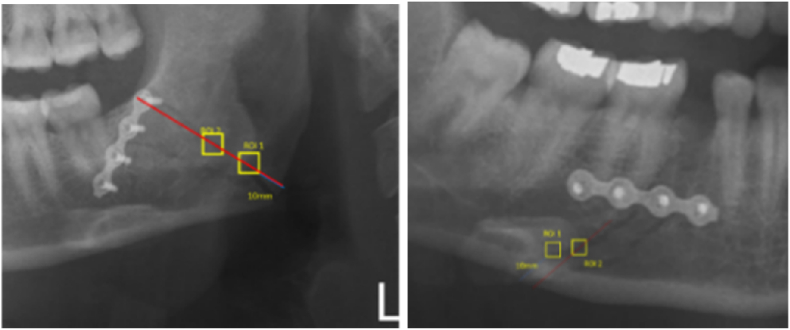
Postoperative ROI determination of mandibular angulus (right); mandibular corpus (left).

The ROI size is 40 × 40 pixels in 1 ROI (Fig. 2) arranged as follows:

a. ROI placement on postoperative panoramic radiograph of mandibular angulus fracture

**Fig. 3:**
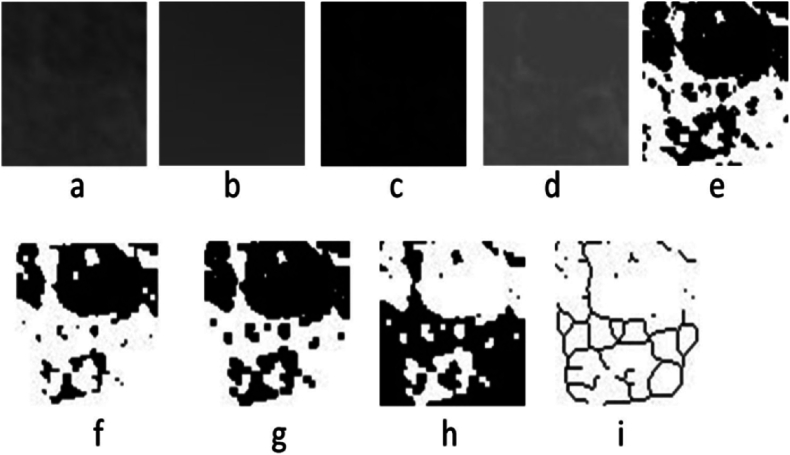
Steps of fractal dimension analysis in ImageJ software.^13^.

The first ROI was located 10 mm above the lower edge of the mandibular angulus at the fracture line. The second ROI is placed in the center area of the superior and inferior border of the mandibular angulus at the fracture line. Measurements were made by determining 2 ROIs in the fracture line. The values of ROI 1 and ROI 2 were summed and then divided by 2 to be the average fractal dimension value of the fracture area.

b. ROI placement on postoperative panoramic radiograph of mandibular corpus fracture

Measurements were made by defining 2 ROIs in the fracture line. The first ROI was located 10 mm above the lower edge of the mandibular corpus in the fracture line. The second ROI lies in the center area of the apical end of the fractured medial tooth and inferior to the mandibular corpus at the fracture line. The values of ROI 1 and ROI 2 were summed and then divided by 2 to achieve the average fractal dimension value in the fracture area.

### 2.3 Fractal dimension analysis in ImageJ software (white and Rudolph, 1999)

The observation area was duplicated in ImageJ software. The bright area of the bone was made blurred with Gaussian Blur Filter. The blurred area was removed from the original image with substract application, and 128Gy tones were added to each pixel with add application. This step can distinguish the trabecular structure of the bone and the bone marrow. The image was converted to black and white with threshold application. Invert application is used to convert the white image to black and vice versa with skeletonize option then the trabecular structure is determined on the bone with lines and ready to be used for fractal dimension analysis. The results of the analysis were calculated with the analyze option, and all applications were performed on the option.^13^

### 2.4 Statistical analysis

Statistical analysis was performed using SPSS 19.0 software. Data were tested for normality with Shapiro-wilk to see that the data were normally distributed, and tested for homogeneity of data variance in more than two groups using the Levene test. As data was normal and homogen, Two-Way ANOVA was used to determine differences in the effect of location and observation time variables on FD values and to determine the interaction between the two. The analysis was continued with the Post hoc-LSD test to analyze significant differences between groups for observations on postoperative 1st day, 2nd week, 8–13th week and >13th week, with p value of <0.05 was considered a significant difference.

## 3 Results

During the study, only 86 of 124 patients with fractures of the angulus and/or corpus mandible (2017–2023) had complete medical record data and postoperative panoramic radiographs. A total of 51 patients were excluded from the study because of incomplete radiographic data (35 patients), beyond the quality requirements (5 patients), treated with 2 mini plates (5 patients) and treated with closed reduction (2 patients) and aged below 18 years (4 patients). As a result, only 35 patients were eligible consisting of 15 patients with fractures of the mandibular corpus, 15 patients with mandibular angulus and 10 patients with mandibular corpus and angulus. Multiple angulus and mandibular corpus fracture groups (10 subjects) were included in each fracture group; therefore, there were 25 samples in each angulus and corpus fracture group. Only patients aged 18–60 years were treated with ORIF using 1 mini plate and MMF archbar (see Table 1).

The subjects involved in the corpus and angulus groups consist of 25 patients aged 18–30 (p = 0.68), gender (p = 1.00) and education level (p = 1.00), favorable fracture line direction (p = 1.00), number of molar teeth (p = 0.44) and nutritional status (p = 0.09 showed no significant difference (p > 0.05) in the study. The length of MMF use (Table 2) in the corpus and angulus groups was not significantly different with an average of 16.1 days and 15.6 days (p = 0.067). The observation time of FD values in H1 (average 1 day), H2 (average 16.08 corpus days; 15.56 angulus days), H3 (average 8.08 corpus and angulus weeks), H4 (average 17.68 corpus days; 17.60 angulus days) was insignificantly different with the respective p value of H1 (1.00), H2 (0.08), H3 (1.00), H4 (0.06).

**Table 1:** Characteristics of subjects.

Patient Group		Corpus fracture (n = 25)	Angulus fracture (n = 25)	p Value
		n(%)	n(%)	
Age (Years)				0.68∗∗
	18–30	15(60%)	18(72%)	
	30–48	7(28%)	5(20%)	
	48–60	3(12%)	2(8%)	
Gender				1.00∗
	Male	12(80)	20(80%)	
	Female	3(20)	5(20%)	
Fracture Line Direction				1.00∗
	Favorable	14(56%)	14(56%)	
	Unfavorable	11(44%)	11(44%)	
Number of Molar Teeth				0.44∗
	0–4	0(0%)	0(0%)	
	5–8	16(64%)	9(36%)	
	9–12	9(36%)	16(64%)	
Socio-Economic Level				1.00∗∗
	< High school	23(92%)	23(92%)	
	> High school	2(8%)	2(8%)	
BMI				0.09∗
<18.5	Thin	8(32%)	11(44%)	
18.5–25	Ideal	15(60%)	10(40%)	
>25–29.9	Fat	2(8%)	4(16%)	
MMF Duration	Mean±SD MMF (Day)	16.1 ± 1.08	15.6 ± 0.87	0.07∗∗

∗Based on *Chi-square* and ∗∗independence Sample *T*-test α = 0.05.

**Table 2:** Length of post-operative observation time.

Observation	Fracture Site (Mean ± SD)		p Value
	Corpus	Angulus	
H1 (postoperative 1st day)	1 day	1 day	1.00
H2 (postoperative 2nd- 8th week)	16.08 ± 1.07 day	15.56 ± 0.87 day	0.08
H3 (postoperative 8th-13th week)	8.08 ± 0.28 week	8.08 ± 0.28 week	1.00
H4 (postoperative >13th week)	17.68 ± 1.55 week	16.64 ± 1.63 week	0.06

Independence Sample *T*-test α = 0.05.

The postoperative condition of the subjects (Table 3) showed that both corpus and angulus fracture groups did not have severe pain, dehiscence, malocclusion and displacement of fracture fragments (fragments appeared stable) in both groups. Mild-moderate pain was found on H1 in all groups (100 %), and on H2 in the angulus fracture group (12 %). Complaints of paresthesia occurred only on H1 of the corpus fracture in 3 subjects (12 %). All complaints disappeared at the next control.

**Table 3:** Postoperative clinical condition of the subjects.

Clinical Evaluation	Observation Time							
	Corpus Fracture				Angulus Fracture			
	H1 n(%)	H2 n(%)	H3 n(%)	H4 n(%)	H1 n(%)	H2 n(%)	H3 n(%)	H4 n(%)
**Vas Scale**								
No Pain (0)	0	25(100)	25(100)	25(100)	0	23(88)	25(100)	25(100)
Mild-Moderate (1–6)	25(100)	0	0	0	25(100)	3(12)	0	0
Severe Pain (7–9)	0	0	0	0	0	0	0	0
**Dehiscence**	0	0	0	0	0	0	0	0
**Paresthesia**	3(12)	0	0	0	0	0	0	0
**Malocclusion**	0	0	0	0	0	0	0	0
**Fragment Shift**	0	0	0	0	0	0	0	0

### 3.1 Fractal dimension (FD) values

Fractal dimension values (Table 4) on panoramic radiography samples using ImageJ software were performed at 4 observation times based on the predetermined observation time of the patient's clinical condition (H1 to H4). Data on FD values in the corpus and angulus groups and observation time groups were normally distributed and homogeneous (appendix).

**Table 4:** Fractal dimension (FD) values observation.

Fracture Site	Observation Time			
	H1	H2	H3	H4
	Mean ± SD	Mean ± SD	Mean ± SD	Mean ± SD
Corpus	1.23 ± 0.029	1.24 ± 0.038	1.32 ± 0.024	1.46 ± 0.031
Angulus	1.17 ± 0.045	1.20 ± 0.051	1.29 ± 0.040	1.35 ± 0.087
p-value	0.000^a^	0.003^a^	0.031^a^	0.000^a^

a Significant based on Fisher's LSD post hoc test.

**Fig. 4:**
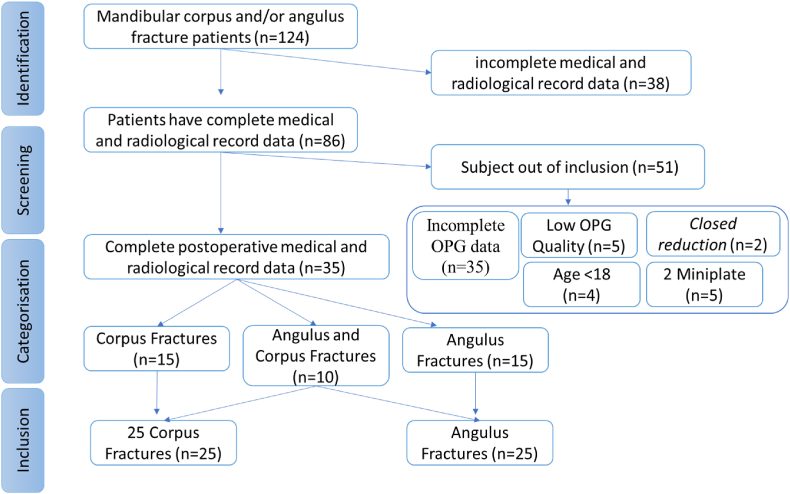
Flowchart of research subject selection.

The results of the Two-Way ANOVA test showed that there was a significant effect between corpus and angulus fracture groups, observation time, and a significant interaction between fracture group and observation time with p value < 0.05 respectively (see Fig. 4) (see Fig. 3).

**Fig. 5:**
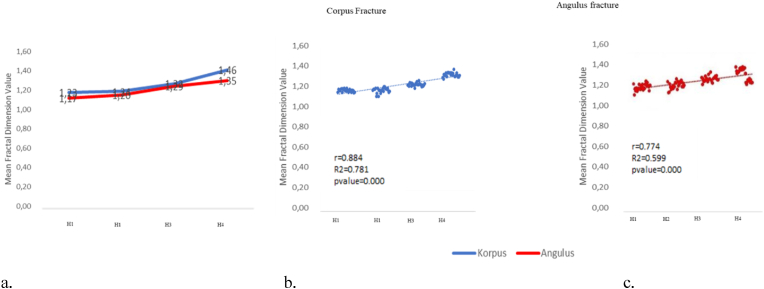
(a) FD value graph shows a significant increase in fractal value until postoperative 17th week; (b and c) scatter plot diagram of correlation of corpus and angulus fracture FD toward the observation time.

The highest fractal dimension value was observed on H4 (postoperative >13 weeks) in both corpus (1.46 ± 0.031) and angulus (1.35 ± 0.087) fracture groups (Table 4), and there was an increase in FD values from H1 (postoperative 1 day) observation to H4 (Fig. 5). Based on the observation time H1 to H4 in both the corpus and angulus fracture groups, it showed an increase in FD values along with the observation time (Fig. 5).

The results of Fisher's LSD Post Hoc Test (Table 4) between corpus and angulus fractures at observation H1 to H4 showed that the FD values of corpus fracture were significantly higher at all observation times when compared to that of angulus fracture with the respective p value: 0.000(H1), 0.003(H2), 0.031(H3), 0.000(H4).

Pearson correlation test showed a relationship between FD values and observation time of H1 to H4 in both corpus (p = 0.000 and r = 0.884), and angulus (p = 0.00 r = 0.774) fracture groups (Fig. 5) until observation time H4 (average observation up to postoperative [17th week). A closer correlation was found in the corpus group. Observations and statistical tests showed that corpus fracture had a better bone healing process in trabeculae formation than mandibular angulus at the observation time.

## 4 Discussion

This study evaluated the healing process of post ORIF mandibular corpus and/or angulus fractures through fractal dimension on panoramic radiographs using ImageJ software as consideration to determine mastication loading time. Fractal dimension was used to assess the pattern of trabeculae formed on panoramic radiographic images in the healing process of mandibular area fractures.^13^ Healing evaluation has so far been carried out clinically by observing the movement of fracture fragments.^9^^,^^10^ Radiographic examination has commonly been done by manually assessing the disappearance of the fracture line, which is subjective to the operator's vision.^11^^,^^18^

Evaluation of the fracture healing process through fractal dimension analysis is used as an addition to the field of radiology for consideration of bone healing evaluation. The analysis is simpler, accurate and non-invasive, and provides quantitative analysis and assessment and assesses trabeculae formation more accurately.^13^^,^^14^ Another method with plain radiographs was presented by Tiwari and Singh (2021). The method used the Radiographic Union Score of Mandible (RUSM) system which assesses cortical healing of fracture lines and bridging callus with OPG and occlusal radiographic modalities. The other analysis was Gray Scale Value (GSV), which is an analysis of bone healing using a ratio (pixel value ratio) that compares the gray value of the gap with healthy bone.^19^^,^^20^ Because this is a crosssectional retrospective study, the researchers cannot manipulate variables and can only analyze data at one predetermined time point, however this is the first study which observed FD in healing procces of corpus and/or angulus mandible fractures.

The FD values in the mandibular corpus fracture group were found greater when compared to those of the mandibular angulus at all observation times from H1 to H4 (average FD value of corpus H1 (1.23); H2 (1.24), H3 (1.32); H4 (1.46)). The results show that the pattern of trabeculae formed in the corpus was more in number when compared to the mandibular angulus (average FD values of angulus H1 (1.17); H2 (1.20), H3 (1.29); H4 (1.35)) at the specified observation time. The finding indicates that the healing process in fractures of the corpus mandible was faster when compared to the angulus.

The difference in the FD values of the H1 observation occurred because before the fracture the corpus area adapted to the occlusion pressure on the masticatory teeth. As a result, the trabeculae in the corpus would be more numerous.^21^ This physiological character suggests that the trabeculae on H1 corpus was larger than the angulus. This indicates that the trabeculae pattern has been radiologically formed despite that theoritically trabeculae is not formed in the healing process.^21^^,^^22^

The effect of immobilization of the fracture area is very important in the healing process, especially in ORIF-treated fractures with 1 mini-plate and archbar MMF to add rigid properties and maintain the occlusion formed (Manzie et al., 2021; Ezhilarasi and Katrolia, 2022).^23^^,^^24^ Removal of immobilization that enables the jaw to move will result in micro-movement, especially in the angulus area. The micro-movement in the angulus is generated by the movement of the main masticatory muscles especially the masseter and pterygoid. It makes the movement greater than the corpus which causes a slowdown in the healing process.^5^^,^^25^ The study used immobilization with 1 mini plate and archbar MMF for 2 weeks in all observation samples. The assessment of FD values was carried out for the longest time on average until 17th week (observation on H4; postoperative >13th week). This is one of the reasons for loading pembebathe angulus later than the corpus. The clinical application of the results can be the basis for MMF release and loading time when corpus and angulus fractures occur together.^25^^,^^26^

It makes sense for this study to find an increase in the fractal dimension (FD) values along with the observation time (postoperative 1st day, 2nd week, 8–13th week and >13th week) in both angulus and corpus fractures with a value of p = 0.00 (p < 0.05). It shows that the increase in FD values was influenced by time (corpus p = 0.000 r = 0.884; angulus p = 0.000 r = 0.774). Similar studies as in Heo et al., 2002, Kang et al., 2012, Colak et al., 2022 and Coban et al., 2022 found the same results but in mandibular BSSO orthognathic surgery patients. The studies found an increase in the FD values in the osteotomy area over time as the bone healing process occurred.

A new trabeculae was formed due to calcification of bone matrix in the central and peripheral areas with active chondroisit in the bone healing process. This stage is commonly called the soft-callus stage which is characterized by periosteal and endosteal calcium deposition and with the growth of new osteoid fingers in postoperative 2nd to 6th week.^22^^,^^27^ The next stage of continuous osteoclast and osteoblast activity and calcium deposition causes immature bone (woven bone) to be transformed into mature (lamellar bone).^22^ This bone was stronger so that osteoclasts could penetrate into the debris tissue in the fracture area and was followed by osteoblasts to fill the gap between the fragments with a new bone. This stage is commonly called the hard-callus stage.^22^, ^27^, ^28^

This hard-callus image will radiologically appear to have changes in the trabecular matrix, while changes in the fracture density occur on average from 10th to 13th weeks, which identifies the peak of hard-callus formation and enters the remodeling phase where trabeculae will fill the medullary cavity.^29^^,^^30^ The afore-mentioned theory was proven in this study as the healing process on postoperative >12 ^th^ week after ORIF showed the phase of remodeling and bone maturation. As a result, the process of forming trabeculae was more visible.^22^

The postoperative clinical condition of the subjects found no dehiscence, malocclusion in all subjects, and fragments appeared stable at the corpus and angulus fracture sites observed. Pain and paresthesia were the most common complaints found on postoperative 1st day, but the complaints disappeared during postoperative 2nd week control. The postoperative pain remained at a mild degree, felt only by 3 subjects (12 %) in group 2 until the second control or 2nd week. Song et al. (2014) found the occurrence of paresthesia (up to 3 years) in the inferior mandibular and mental nerve alveolaris by 13 % of the samples with ORIF 2 mini plates. This finding shows that the installing 2 mini plates is more prone to paresthesia due to inflammation or trauma to the involved nerve area.

This is the first study to evaluate bone healing in trabeculae structure of mandibular corpus and angulus fracture patients with fractal dimension analysis as consideration to determine mastication loading time. The study has limitation in a number of samples.

## 5 Conclusion

Post ORIF observation of corpus fractures shows greater trabeculae formation values which indicate faster healing when compared to that of mandibular angulus fractures. This fractal dimension analysis assessment can be used to evaluate the bone healing process.

## Consent to participate and consent for publication

Not Aplicable.

## Patient consent

This study uses secondary data that obtained from medical records searches.

## Ethical approval

Not Aplicable.

## Availability of supporting data

The data set ad/or analysed during the current study are available from the corresponding author on reasonable request.

## Ethical clearance

Ethical eligibility of the study was approved by the ethics committee of Faculty of Dentistry of the Mada University (32/UN1/KEP/FKG-RSGM/EC/2024).

## Source of funding

Self funded

## Declaration of competing interest

The authors have no conflicts of interest to disclose.
